# Dentures Used for Rehabilitation of Mastication After Loss of Teeth Maintain Proper Biomechanical Properties of Masseter Muscles—A Comparative Study

**DOI:** 10.3390/clinpract15020032

**Published:** 2025-02-10

**Authors:** Sebastian Szajkowski, Jarosław Pasek, Grzegorz Cieślar

**Affiliations:** 1 Faculty of Medical Sciences, Warsaw Medical Academy of Applied Sciences, 8 Rydygiera St., 01-793 Warsaw, Poland; sebastianszajkowski@wp.pl; 2 Collegium Medicum im dr Władysława Biegańskiego, Jan Długosz University in Częstochowa, 13/15 Armii Krajowej St., 42-200 Częstochowa, Poland; 3 Department of Internal Medicine, Angiology and Physical Medicine, Faculty of Medical Sciences in Zabrze, Medical University of Silesia in Katowice, 15 Stefana Batorego St., 41-902 Bytom, Poland; cieslar1@o2.pl

**Keywords:** dentures, masseter muscle, bite force, stiffness, myotometry

## Abstract

**Background**: Various types of dentures are applied in the treatment of missing teeth. The research carried out so far has proven that both the absence of teeth and the replacement of missing teeth with dentures affect the functional condition of masseter muscles. The purpose is, thus, to find out how the tone, elasticity, and stiffness of masseter muscles change after prosthetic treatment. **Material and methods**: The material for the study consisted of 137 individuals, who were divided into group 1, with dentures (n = 87) and undergoing prosthetic treatment, and group 2, without dentures (n = 50) with teeth preserved, and not undergoing prosthetic treatment. Additionally, group 1 was divided into subgroups, in accordance with the type of prosthetic treatment applied: group 1a—removable complete dentures (n = 14), group 1b—removable partial dentures (n = 48), group 1c—fixed partial dentures (n = 22), group 1d—fixed complete dentures (n = 3). The biomechanical properties of masseter muscles, namely: tone, stiffness, and elasticity were assessed by means of myotonometry. The measurements were taken under muscle relaxation and under maximum muscle contraction. **Results**: The values of the parameters of tone, stiffness, and elasticity were higher in group 2, without dentures, in comparison with group 1, with dentures, yet they did not differ with statistical significance. The type of prosthesis used affected the results obtained. Masseter muscles demonstrated the best biomechanical parameters in the case of applying fixed partial dentures. **Conclusions**: Prosthetic replacement of missing teeth contributes to maintaining the good condition of masseter muscles, which has been confirmed by the results of myotonometric measurements taken.

## 1. Introduction

The purpose of reconstruction and prosthetic rehabilitation of the stomatognathic system in cases of partial or complete loss of dentition is to provide functional and aesthetic restoration of missing teeth by means of various types of prostheses, taking into account the broadly understood prophylaxis, at the same time. The following contribute to the disorder of food mastication function: loss of teeth and accompanying weakness, as well as atrophy of muscles composing the mastication apparatus. Due to the loss of teeth, the bite force and mastication efficiency are reduced [1]. The masseter muscle is the main muscle that lifts the lower jaw. Three muscles cooperate in this activity: the pterygoid muscle, which is responsible for about 21% of the total jaw-closing muscle strength, the temporal muscle is responsible for about 36% of that strength, and the masseter muscle which is responsible for some 43% of such strength [2].

Various indicators and parameters of muscles are used to objectively assess the functional condition of masseter muscles. Among the most important ones is the maximum bite force that a muscle can generate. Studies have shown that the bite force is proportional to the cross-sectional area (CSA) of the muscle [3]. Previous studies focused primarily on the influence of age on the size of the masseter muscle and showed its significant reduction which was age-related [4]. However, more recent evidence suggests that the reduction in chewing muscle size is not directly the result of the aging process as such, but is rather caused by age-related local and systemic diseases, resulting in tooth loss and a reduced amount of functional tooth units remaining available in the mouth [5]. Other studies were conducted to determine the effect of restoring occlusal function/bite force by means of prostheses upon the mastication efficiency in toothless patients. Some of them have shown an increase in muscle thickness and improvement in chewing function, up to a level close to that present in individuals with full natural dentition [6,7]. Other studies [8], on the contrary, have shown a significant reduction in muscle thickness and chewing performance in people with artificial dentition, in comparison with persons with natural dentition. It has been suggested that continuous reduction of muscle thickness and performance is associated with tooth loss and altered functional requirements, which artificial dentition is unable to make up for.

Previous studies provide inconsistent data concerning the impact of prosthetic treatment upon masseter muscles. Thus, this is the basis for exploring this problem further. While previous studies have used bite force measurement and CSA to assess the condition of masseter muscles in people with tooth loss and undergoing prosthetic treatment, none of those studies evaluated the biomechanical properties of the muscles that have direct influence upon the biomechanics of chewing apparatus, including bite force [3]. Such assessment is made possible by applying myotonometry, which is a promising and objective method of making a quantitative assessment of the biomechanical properties of muscles and tendons. It can successfully replace subjective palpation examinations and has been widely described in the literature as reliable, being a method which ensures high repeatability of measurements. In addition, my tonometer measurements are easy to perform and do not take much time. The MyotonPRO device (Myoton AS, Tallinn, Estonia) used in the study has been demonstrated to be a reliable device for testing the biomechanical parameters of masseter muscle [9,10]. In addition, the superficial location of the masseter muscle provides easy access to carry out measurements. Results concerning specific parameters: tone, elasticity, and stiffness obtained in myotonometric examinations carried out in vivo allow us to assess the functional condition of the muscles. They are used both in diagnostics and in the assessment of treatment results.

## 2. Aim of the Study

The comparative study reported here has aimed to investigate the biomechanical properties of masseter muscles using myotonometry, and subsequently compare the results between persons with missing teeth supplemented with dentures and individuals with natural dentition, not requiring prosthetic treatment.

## 3. Material and Methods

### 3.1. Study Design

**Figure 1:**
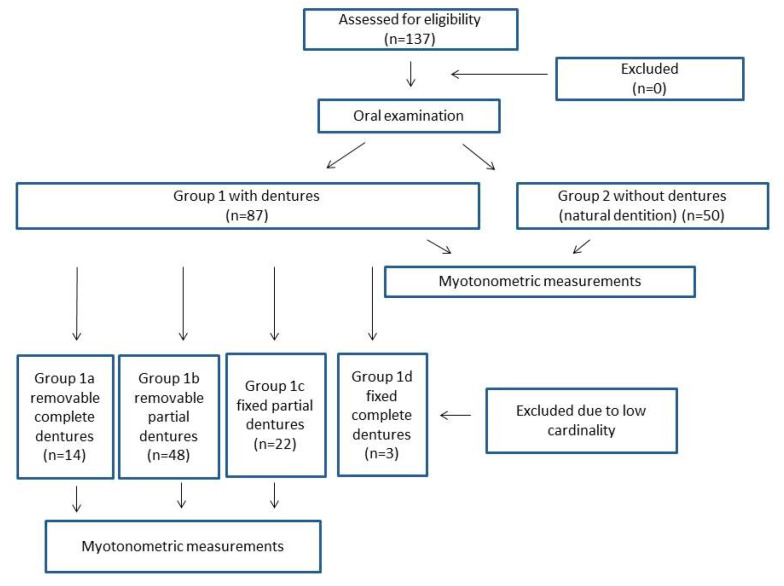
Study design.

137 individuals (77 females and 60 males) were qualified for the study. The study material was divided into 2 groups. Group 1, with dentures (n = 87; 46 females and 41 males), and group 2, without dentures (natural dentition) (n = 50; 27 females and 23 males). The age range of study participants was from 55 to 70 years of age. Additionally, group 1 was divided into subgroups, in accordance with the type of prosthetic treatment used: group 1a—removable complete dentures (n = 14), group 1b—removable partial dentures (n = 48), group 1c—fixed partial dentures (n = 22), group 1d—fixed complete dentures (n = 3); (Figure 1). The sequence of procedures was as follows: 1—oral examination, 2—myotonometric measurement in group 1 and group 2, 3—myotonometric measurement in groups 1a,1b, and 1c. Group 1d has been excluded, due to its low cardinality.

The study comprised patients who met the inclusion and exclusion criteria specified (Table 1).

### 3.2. Oral Examination and Myotonometric Measurements

All oral examinations were performed on the participants by a single-trained dental hygienist. Each participant’s dentition was examined. Myotonometric measurements of masseter muscles were performed in a sitting position with the head held in a neutral position. Constant conditions were provided for taking the measurements, namely: constant ambient temperature of 22 °C, 15 min rest observed by the subject before taking the measurements; participants were instructed to refrain from eating food for one hour preceding the measurements. Myotonometric measurements were first taken on relaxed muscle and were performed on both sides. That was followed by taking the measurements under maximum muscle contraction [9]. The measurements were repeated twice. The measurement results were averaged separately for the right and left side and, respectively, for measurements taken under muscle relaxation and maximum muscle contraction. During the examination, the coefficient of variation (CV) of each test result was noted, and if the CV exceeded 3%, the test was repeated once again [13].

**Figure 2:**
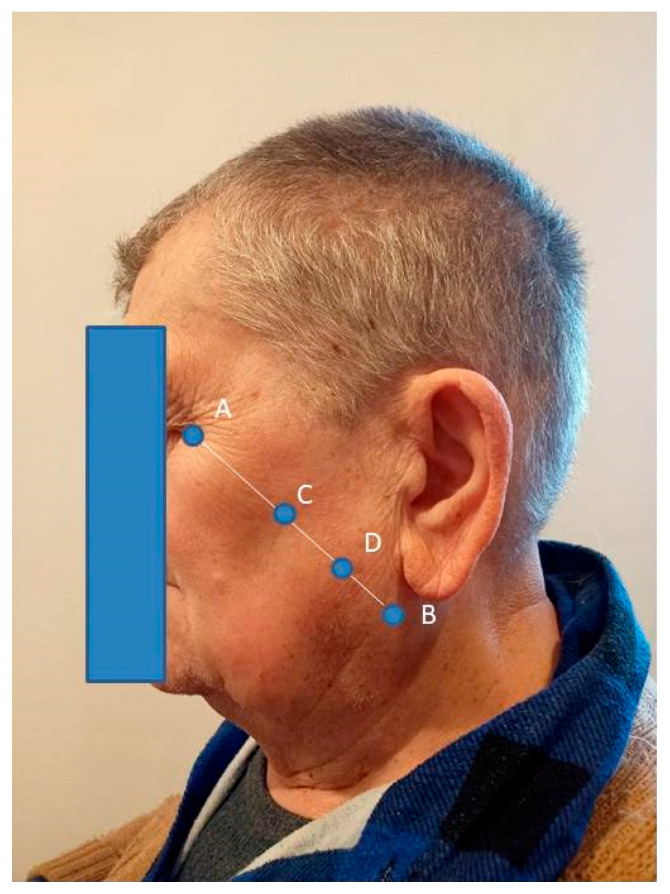
A measurement point of the masseter muscle. A—the participant’s eye corner, B—the mandibular angulus, C—the point of intersection between the line (A, B) and the zygomatic bone, D—the middle point of the line (B, C) set as the measurement point of the masseter muscle.

For the purpose of determining the measurement point corresponding to the largest cross-section of the muscle, a straight line was connected from the participant’s eye corner (A) to the mandibular angulus (B), while the point of intersection between the line (A, B) and the zygomatic bone was defined as C. The middle point of the line (B, C) was set as the measurement point of the masseter muscle (D) (Figure 2), in accordance with the study by Hara et al. [3]. The probe of MyotonPRO (Myoton AS, Tallinn, Estonia) was placed at the point predetermined earlier and marked. After the stabilization of the device, an automatic preload of 0.18 N was applied, and an automatic mechanical impulse was initiated for 15 ms at a predetermined force of 0.4 N. The following were calculated: frequency (F) [Hz], which characterizes tone; stiffness (S) [N/m], indicating the ability of the tissue to resist an external force that modifies its shape; and decrement (D) [log], which characterizes elasticity (the ability of a muscle to recover its shape after being deformed), the lower its value the higher the elasticity [14].

The outcomes were interpreted as follows: the higher the values of (F) and (S), the greater the tone and stiffness of tissue; the lower the value of (D), the lower the dissipation of mechanical energy during oscillation, and the higher the elasticity of tissue. Intra-rater reliability was good (ICC = 0.78) and inter-operator reliability was excellent (ICC = 0.95) for assessing masseter muscle stiffness with the use of MyotonPRO [9]. All the measurements were performed by an experienced physiotherapist.

### 3.3. Statistical Analysis

The Statistica 13 package (Statsoft, Kraków, Poland) was used to analyze the data. The distribution of the data was verified using the Shapiro–Wilk test. The results were presented using the median, lower Q1, and upper Q3 quartile, due to the non-normal distribution of data. Mann Whitney U test was used to examine the changes in the examined.

Variables between 2 groups, and Kruskal Wallis Test followed by post-hoc Dunn’s test for comparison of parameters between 3 groups. Statistical significance was set at *p* < 0.05. A priori power analysis was conducted with the G*power software (version 3.1.9.7; Heinrich-Heine-Universität Düsseldorf, Düsseldorf, Germany; (http://www.gpower.hhu.de) (accessed on 15 December 2024) [15]. Two independent groups (non-parametric) with an effect size of 0.5, α = 0.05, and 1-β = 0.8, allocation ratio N2/N1 = 2, gave a power of 0.8 and the total sample size of 118 subjects, the sample size of group 1 = 39 subjects and of group 2 = 79 subjects.

## 4. Results

The average age in group 1 was 65.97 ± 7.01 years of age, in group 2 it amounted to 63.68 ± 5.91 years. The difference was not of statistical significance (*p* = 0.085). The average BMI value amounted to 24.61 ± 4.17 kg/m^2^ in group 1, and to 23.83 ± 3.72 kg/m^2^ in group 2. The difference was not statistically significant (*p* = 0.324) either. The number of women and men did not differ statistically significantly either in group 1 (*p* = 0.644) or in group 2 (*p* = 0.713). The median (Q1–Q3) for missing teeth in group 1b amounted to 11 (9–13) whereas in group 1c it amounted to 9 (9–11). The difference noted was statistically significant (*p* = 0.004).

Statistical analysis of the results of the myotonometric measurements carried out indicated that values of tone, stiffness, and elasticity of masseter muscle were higher in group 2, without dentures (natural dentition), in comparison with group 1, with dentures, but the results did not differ at a statistically significant level. This concerned all the measurements taken, that are under muscle relaxation as well as during forceful biting, on both sides (Table 2).

In group 1, statistically significant differences have been detected in the parameters examined, those differences existed between various types of prosthetic restoration applied. Statistically significant differences concerned the values of tone, stiffness, and elasticity measured on the left and right side during forceful biting (Table 3). The values of tone and stiffness were the highest, with elasticity being the lowest in group 1c, with fixed partial dentures. The values of these parameters were lower in group 1b, with removable partial dentures, and the lowest in group 1a, with removable complete dentures. Multiple comparisons showed statistically significant differences for the parameter F (flex) on the left side between groups: 1a vs. 1c (*p* = 0.002) and 1b vs. 1c (*p* < 0.001), for parameter S (flex) on the left side between groups: 1a vs. 1c (*p* < 0.001) and 1b vs. 1c (*p* < 0.001), for parameter D (flex) on the left side between groups: 1a vs. 1b (*p* = 0.002) and 1a vs. 1c (*p* = 0.009), for parameter F (flex) on the right side between groups: 1a vs. 1c (*p* = 0.007) and 1b vs. 1c (*p* = 0.086), for parameter S (flex) on the right side between groups: 1a vs. 1c (*p* < 0.001) and 1b vs. 1c (*p* = 0.024), and for parameter D (flex) on the right side between groups: 1a vs. 1b (*p* = 0.061) and 1a vs. 1c (*p* = 0.025). There were statistically significant differences (*p* > 0.05) concerning the values of tone, stiffness, and elasticity measured on the right and left side, at the relaxation of masseter muscles.

In 55 cases the dominating side involved in chewing in the case of group 1 was the right side, and in 32 cases it was the left side. In group 2, in 30 cases the side dominating for chewing was the right side, and in 20 cases it was the left side. Neither in group 1 nor in group 2 statistically significant differences (*p* > 0.05) were found in the studied parameters, as regards the dominating and not dominating side for chewing.

## 5. Discussion

Masseter muscles significantly determine the proper functioning of the chewing apparatus [16]. The main factor affecting the chewing ability is the number and location of teeth remaining in the jaws [17]. However, chewing efficiency should not be assessed solely on the basis of the number of remaining teeth, because the chewing movement is coordinated by the functional occlusion system, which comprises teeth, temporomandibular joints, and masticatory muscles [18]. Dysfunction of any of the above components results in reduced efficiency of chewing food. In our study, not only premolars and molars were taken into account as teeth mainly involved in mastication, but we also assessed all other remaining teeth as well as artificial dentures, in order to arrive at the most accurate functional assessment of masseter muscles. In the study, we used the MyotonPRO device to assess the biomechanical properties of masseter muscles in people undergoing prosthetic treatment, as well as those who do not require such treatment. Research findings regarding the biomechanical properties of masseter muscles indicate that values of the parameters of tone, stiffness, and elasticity turned out to be higher in the group not using dentures (with natural dentition) in comparison with individuals from the group with dentures, yet the differences were not of statistical significance. This means that the prosthetic replacement of missing teeth contributes to preserving masseter muscles in good condition, with biomechanical parameters remaining close to those measured in healthy individuals.

The above has also been confirmed in the study [7] which has shown a significant increase in the thickness of the masseter muscles and temporal muscles, both in contraction and at rest, measured by means of ultrasonography, after implantation of fixed artificial dentures supported on implants in toothless patients, reaching a similar muscle thickness as in case of people with dentition, as early as after six months. It has also been confirmed by the study [19], in which individuals with TMD and dentulous ones, presented with higher electromyographical activity than the individuals with TMD and lacking posterior teeth. In another study, it has been proven that extensive bite force loss might be related to diurnal masticatory muscle parafunction and diurnal phasic masticatory muscle activity, which was characteristic in patients with progressive bite collapse [20]. Hara et al. [3] conclude that masseter muscle stiffness possibly reflects a force generated by the masseter muscle during forceful biting, and this parameter could be effective in assessing tooth loss, as well as the index of masseter muscle strength when evaluating maximum bite force.

The authors managed to find a single study employing myotonometry for the assessment of muscles constituting the mastication apparatus in case of missing teeth [12]. This study showed that the tone and flexibility of the masseter muscles decreased as the masticatory function deteriorated. This suggests that masseter muscle atrophy progresses in older adults with reduced chewing performance. Another study [21] showed that the cross-sectional area (CSA) of the masseter muscles decreases with age as a result of reduced activity caused by increasing difficulty with chewing and crushing food, while the study of Murakami et al. [22] has shown that reduction of muscle mass significantly reduces the ability to chew.

Our study also showed statistically significant differences in the range of parameters studied, occurring between different types of dental prosthetic restoration. Tone and stiffness values were the highest and flexibility was the lowest in the group with fixed partial dentures. The values of the above parameters were lower in the group of individuals using removable partial dentures, and the lowest in the group of persons with removable complete dentures. Statistically significant differences noted were related to the parameters measured both on the left and right side, but only during forceful biting. No significant differences were revealed in relaxation. Thus, masseter muscles show the best biomechanical parameters when fixed partial dentures are used. They are most similar to those observed in healthy individuals who do not require artificial dentures. However, it should be kept in mind that statistically significantly lower quantities of missing teeth were observed in this group (median: 9 vs. 11) which may influence the results obtained. To the best of our knowledge, this topic has so far not been considered in the literature to which our observations could be referred, as far as values of myotonometric parameters measured in relation to artificial dentures apply.

Aging influences the thickness of masseter muscles, the morphology of dental arch occlusion, and bite force. The thickness of masseter muscles is related to facial morphology and anthropometric variables such as the thickness of the alveolar ridge or the width of the dental arch of the jaw [3,23]. The influence of age and gender was also demonstrated [24]. That is why, according to the inclusion criteria, ultimately we recruited participants not significantly differing between groups in terms of age, BMI values, or the number of women and men involved in the study.

The measurement of masseter muscle stiffness by means of myotonometry is correlated with muscle strength [9]. There are numerous studies evaluating the strength of the muscles involved in biting and chewing performance after prosthetic treatment, which is directly dependent upon the biomechanical properties of masseter muscles [3,25,26]. Muscle stiffness assessed in myotonometric examination during maximum muscle contraction is a recognized and indirect measure of muscle strength [3,9]. The stiffness of the muscle increases linearly with the strength of contraction. Therefore, it is assumed that stiffness reflects the strength generated by the muscle in the course of forceful biting. As the muscle stiffness increases, the tone also increases, whereas elasticity decreases. The increase in cross-sectional area (CSA) is proportional to the increase in tone. The difference in the thickness of the masseter muscle is considered to be a good indicator of tone. The measurements of stiffness and tone have the potential to assess the force generated as well as the activation of masseter muscle during forceful biting [27,28]. It was shown that the thickness of the masseter muscles corresponds with the bite force, which is in congruence with the above findings [27].

MyotonPRO is easy to operate and used for examining the biomechanical parameters of muscles. It is a reliable method for quantifying the stiffness of the masseter muscle and monitoring its changes under different contraction conditions. The results of measurements taken using this device, due to the high repeatability of the results obtained, may exceed the value of examinations with the application of EMG, which device, in turn, is characterized by limitations due to the fact that the electrical signal is prone to numerous disturbances [29]. Moreover, myotonometry is many times cheaper than ultrasound examination [7] as well as shear-wave elastography (SWE) [30], also used for examining muscle stiffness. It can undoubtedly be a method of choice or a valuable complementary method for other examinations monitoring the condition of muscles making up the mastication apparatus.

## 6. Limitations of the Study

Our study contains some limitations. The study provides an answer to the question concerning the change in biomechanical parameters of masseter muscles as a result of applying dentures used to replace missing teeth, in comparison with healthy individuals. However, the study does not provide the answer to the question concerning the impact of the use of dentures on the biomechanical properties of masseter muscles in comparison with individuals who happen to have many missing teeth, and who require prosthetic treatment but are not treated. Despite the fact that myotonometric examinations have been carried out in one standardized location in the masseter muscle, they may not have been representative of the whole muscle. The maximum masseter muscle contraction was defined subjectively. Moreover, the maximum bite force is related to the overall physical strength, which factor could have influenced the research results obtained.

## 7. Conclusions

Prosthetic replacement of missing teeth contributes to maintaining the good condition of masseter muscles. In such cases, the biomechanical parameters of masseter muscles turn out to be similar to those observed in healthy individuals. The type of prosthesis used affects the functional state of the masseter muscles.

## Figures and Tables

**Table 1:** Inclusion and exclusion criteria in groups.

	Inclusion Criteria	Exclusion Criteria (for Both Groups)
Group 1	Necessary condition: prosthesis of 3 or more teeth from the canines to the molars on at least one side, additionally, provided that the necessary condition has been met, possible prosthesis of 1, 2, or 3 teeth from canine teeth to incisors on one side at least, partial or complete dentures, fixed or removable dentures, remaining teeth are referred to as all teeth capable of chewing, including healthy teeth, teeth with fillings, and teeth affected by caries, normal craniofacial morphology, prosthetic treatment provided for at least 5 years [11,12].	Occurrence of craniofacial anomalies, systemic diseases affecting muscles and chewing function, malocclusion, periodontal diseases, para-functional habits including bruxism and clenching, temporomandibular joint disorders (e.g., trismus and pain during jaw movement), BMI < 17 kg/m^2^ and BMI > 30 kg/m^2^.
Group 2	individuals with 28 natural teeth in addition to third molars were included, missing teeth not replaced by prosthesis: maximum 1 on each side, the remaining teeth are referred to as all teeth capable of chewing, including healthy teeth, teeth with filling, and teeth affected by caries, normal craniofacial morphology [7].	

**Table 2:** The scores concerning myotonometric outcomes in group 1 and group 2, with statistical analysis.

	Group 1, with Dentures (n = 87)		Group 2, Without Dentures (Natural Dentition) (n = 50)		
	Median	Q1–Q3	Median	Q1–Q3	*p*-Value
F (relax) left	16.7	14.8–18.6	17.2	13.9–17.9	0.302
S (relax) left	348	318–361	353	338–380	0.356
D (relax) left	2.2	2–2.56	2.7	2.35–2.77	0.245
F (flex) left	25.2	22.6–27	26.1	24.5–27.9	0.209
S (flex) left	610	510–701	662	559–701	0.504
D (flex) left	1.69	1.4–2.11	1.77	1.35–1.94	0.698
F (relax) right	16.2	14.9–17.6	16.8	15.1–18.5	0.574
S (relax) right	358	340–387	363	330–381	0.299
D (relax) right	2.23	2.02–2.54	2.37	2.03–2.59	0.582
F (flex) right	25.6	23.7–27.2	26.1	24.8–27.6	0.355
S (flex) right	614	543–700	623	577–729	0.519
D (flex) right	1.64	1.34–1.99	1.8	1.4–2.35	0.315

Abbreviations used: F—Frequency [Hz], S—Stiffness [N/m], D—Decrement [log].

**Table 3:** The scores concerning myotonometric outcomes in groups 1a, 1b, and 1c, with statistical analysis.

	Group 1a, Removable Complete Dentures (n = 14)		Group 1b, Removable Partial Dentures (n = 48)		Group 1c, Fixed Partial Dentures (n = 22)		
	Median	Q1–Q3	Median	Q1–Q3	Median	Q1–Q3	*p*-Value
F (relax) left	16.35	13.4–17.5	17.55	14.8–18.6	16.65	15.2–18.7	0.398
S (relax) left	338.5	299–378	351	335–402	351	334–378.5	0.304
D (relax) left	2.19	1.91–2.48	2.21	2.05–2.58	2.34	1.96–2.68	0.596
F (flex) left	21.4	19.1–25.8	22.75	21.1–24	26.7	25.15–27.35	**<0.001**
S (flex) left	473	423–512	530.5	498–605	682.5	610.5–715	**<0.001**
D (flex) left	1.5	1.22–1.88	1.89	1.63–2.32	1.9	1.69–2.19	**0.002**
F (relax) right	15.25	13.2–17.2	15.85	14.9–17.2	16.3	15.25–17.95	0.166
S (relax) right	337	307–383	365	324–389	360	343–385	0.195
D (relax) right	2.24	2.02–2.51	2.24	2.09–2.5	2.28	1.91–2.58	0.909
F (flex) right	21.8	18.4–26	24.55	22.1–26.8	26.1	24.85–27.3	**0.015**
S (flex) right	483	405–572	558.5	506–648	637.5	599.5–703	**0.007**
D (flex) right	1.51	1.33–1.83	1.82	1.63–2	1.85	1.4–2.47	**0.034**

Abbreviations used: F—Frequency [Hz], S—Stiffness [N/m], D—Decrement [log].

## Data Availability

The datasets used and/or analyzed during the current study are available from the corresponding author upon reasonable request.
